# Detection of *Anaplasma capra* in Patients with Suspected Crimean-Congo Hemorrhagic Fever, Turkey, 2022

**DOI:** 10.3201/eid3210.251049

**Published:** 2026-10

**Authors:** Kursat Altay, Nazif Elaldi, Ufuk Erol, Binnur Koksal, Omer Faruk Sahin, Ayse Nur Pektas, Seyit Ali Buyuktuna, Murtaza Oz, Yasemin Cakır Kıymaz, Tuba Nur Tasseten, Husnu Furkan Sakar, Mehmet Bakir

**Affiliations:** Sivas Cumhuriyet University Faculty of Veterinary Medicine, Sivas, Turkey (K. Altay, U. Erol, O.F. Sahin, H.F. Sakar); Sivas Cumhuriyet University Faculty of Medicine, Sivas (N. Elaldi, S.A. Buyuktuna, M. Oz, Y. Cakır Kıymaz); Sivas Cumhuriyet University Faculty of Health Sciences, Sivas (B. Koksal); Sivas Cumhuriyet University Advanced Technology Application and Research Center, Sivas (A.N. Pektas); Sivas Cumhuriyet University Institute of Science, Sivas (T.N. Tasseten); Medicana Sivas Hospital, Sivas (M. Bakir).

**Keywords:** *Anaplasma capra*, Crimean-Congo hemorrhagic fever, *gltA*, *groEL*, genotyping, tick-borne infection, vector-borne infections, viruses, bacteria, Turkey, Türkiye

## Abstract

*Anaplasma capra* is a recently discovered bacterial tickborne pathogen that was first identified in goats in 2012 and later in humans in China in 2015. We detected and genetically characterized *A. capra* in adult patients with suspected Crimean-Congo hemorrhagic fever (CCHF) in Turkey during the 2022 outbreak. We screened all patients for *A. capra* by using nested PCR targeting the *gltA* and *groEL* genes. We confirmed CCHF by molecular and serological tests. Of 263 patients, 6 (2.28%) were positive for *A. capra*. Sequence analysis revealed 84.16%–100% *gltA* gene similarity and 90.24%–100% *groEL* gene similarity with GenBank entries. Phylogenetic analysis confirmed all *A. capra* gene sequences belonged to genotype 1. Our results demonstrate clinical evidence of genotype 1 *A. capra* infection in humans. Our findings highlight the need for clinicians to consider *A. capra* in the differential diagnosis of CCHF-like symptoms, especially in endemic areas.

Ticks are hematophagous ectoparasites of the subclass Acari, comprising ≈900 species worldwide. Ticks act as vectors for >200 human and animal pathogens, including bacteria such as the rickettsiae and viruses (*1*). The incidence and effect of tickborne diseases have risen globally because of environmental and weather changes (*2*). Tickborne infections impose a substantial global burden, with hundreds of thousands of human cases reported annually; Lyme disease alone is estimated to cause ≈450,000 cases each year in the United States (*3*,*4*). Turkey, situated at the crossroads of Europe and Asia, is epidemiologically critical for zoonotic infections, with 107 zoonoses reported and 21 classified as high priority by European health authorities (*5*,*6*). In Turkey, ticks serve as major vectors for various zoonotic diseases, including anaplasmosis and Crimean-Congo hemorrhagic fever (CCHF) (*5*–*7*).

Currently, CCHF is the most prevalent tickborne viral disease in humans and is endemic in parts of Africa, Asia, the Middle East, and southeastern Europe. Turkey has reported the highest number of CCHF cases globally (*8*–*10*). The first CCHF cases were documented in 2002 in the Middle Anatolia–Black Sea region, a hyperendemic area for the disease, and ≈1,000 cases have been reported annually since (*10*,*11*). The causative agent, CCHF virus (CCHFV), a member of the Nairoviridae family within the Bunyavirales order, is primarily transmitted by *Hyalomma marginatum* ticks. No licensed vaccine or effective antiviral treatment is available for CCHF (*12*). The disease typically manifests nonspecific symptoms such as fever, fatigue, myalgia, and gastrointestinal complaints but can progress to severe hemorrhagic manifestations. Case-fatality rates (CFRs) vary geographically, reaching up to 30% in some regions; in Turkey, the CFR is ≈5% (*12*,*13*).

Anaplasmosis, caused by *Anaplasma marginale*, *A. centrale*, *A. phagocytophilum*, *A. bovis*, *A. ovis*, and *A. platys* bacteria, is one of the consequential tickborne zoonotic bacterial infections and is transmitted to vertebrate hosts mainly by *Ixodid* species ticks. *A. phagocytophilum* is the etiologic agent of anaplasmosis in humans, dogs, horses, and ruminants (*14*). Advances in nucleic acid amplification tests and phylogenetic studies in comparing the DNA sequences of pathogens have enabled the detection of new genotypes and species of tickborne pathogens, revealing the necessity of reinterpreting the etiology of cases (*14*–*18*). In 2012, a study designed to detect *Anaplasma* bacteria in goats (*Capra aegagrus hircus*) in northern China revealed a new *Anaplasma* bacterial gene sequence (*19*). In 2015, the same gene sequence was detected in humans in China and was subsequently named *A. capra.* *A. capra* DNA was detected by nested PCR for the *gltA* gene in 28 (5.9%) of 477 patients who had a tick-bite history (*20*). Symptoms manifested as influenza-like illness, including fever, headache, malaise, dizziness, chills, gastrointestinal symptoms, and elevated hepatic enzyme concentrations. Leukopenia and thrombocytopenia were reported only in a limited number of cases (*20*).

Of note, ≈24% of patients with suspected CCHF in Turkey were found to be negative for CCHFV by molecular and serological tests, suggesting alternative tickborne etiologies (*21*). In addition, *A. capra* has been detected in domestic ruminants and water buffalo in the Middle Anatolia–Black Sea region of Turkey, supporting its tickborne nature and suggesting possible human infection in the same area (*15*,*18*). Our aim is to investigate the presence and molecular characteristics of *A. capra* in hospitalized patients with suspected CCHF in a hyperendemic region of Turkey.

## Material and Methods

### Study Design and Patient Selection

This retrospective study included 263 adult patients (>18 years of age) who were hospitalized with suspected CCHF at the clinical ward of the Department of Infectious Diseases and Clinical Microbiology, Sivas Cumhuriyet University Hospital (SCUH), Sivas, Turkey, during a CCHF outbreak in 2022. SCUH is a tertiary referral hospital designated by the Turkish Ministry of Health for CCHF case management, serving a population of ≈1.5 million and located in the hyperendemic Middle Anatolia region. At admission, patients underwent a thorough physical examination and clinical history assessment by ward clinicians.

We included patients in our study on the basis of previously established case definitions (*22*). Study patients had >2 clinical manifestations: fever, headache, diffuse body pain, arthralgia, weakness, diarrhea, or bleeding. In addition, patients had to meet >1 criteria: residence in or recent travel to a CCHF-endemic region within the previous 2 weeks, a history of tick bite, thrombocytopenia (<1.5 × 10^5 ^platelets/mm^3^), or leukopenia (<4 × 10^3^ leukocytes/mm^3^).

### Sample Collection and CCHF Diagnosis

Three blood samples were collected from each patient. One sample was sent to the SCUH central laboratory for routine biochemical and hematological analysis; a second, 2 mL EDTA-treated (BD, https://www.bd.com) whole blood sample, was processed at the university’s Biosafety Level 2 CCHF Research Laboratory for DNA extraction; and the third sample was forwarded to the Turkish Ministry of Health’s Central Reference Virology Laboratory in Ankara for confirmation of CCHF via molecular and serologic testing.

The EDTA-treated whole blood samples were anonymized and stored in a deep freezer at −80°C) until DNA extraction. CCHF diagnosis was confirmed by a positive commercial CCHFV qualitative reverse transcriptase PCR (qRT-PCR) test kits (Bioeksen, https://www.bioeksen.com) or commercial CCHFV IgM indirect immunofluorescence test kits (Euroimmun, https://www.euroimmun.com).

We obtained epidemiologic data, including age, sex, place of residence (village, district, and city), occupation, tick exposure history, date of symptom onset, hospitalization, and sample collection via face-to-face interview records. We retrieved clinical findings, laboratory values, and outcomes (fatal or nonfatal) from the hospital’s electronic medical record system. All patients were managed by the same clinical team and received standard supportive care, including fluid resuscitation, blood product transfusions (fresh frozen plasma, packed red cells, and apheresis platelets), and cardiovascular and respiratory support as needed (*22*). Oral ribavirin therapy was administered at the discretion of the clinician according to a standard protocol (30 mg/kg loading dose, then 15 mg/kg/6 h for 4 d, then 7.5 mg/kg/8 h for 6 d). Patients were discharged when afebrile, without bleeding, with platelet counts >100,000/mm^3^, and with unremarkable coagulation parameters.

Routine microbiological testing included blood and urine cultures at admission. Complete blood count, serum biochemical tests (aspartate aminotransferase, alanine aminotransferase, creatine phosphokinase, lactate dehydrogenase), and coagulation parameters (prothrombin time and activated partial thromboplastin time) were assessed at baseline and monitored as needed during hospitalization.

### *Brucella* spp. Bacteria Testing

In the clinical ward, testing for brucellosis is routinely performed on samples from patients with suspected CCHF (*23*). Diagnostic procedures include the Rose Bengal test, Wright serum agglutination test, Coombs antiglobulin test, and automated blood cultures. Clinicians interpreted the Rose Bengal test as positive or negative on the basis of visible agglutination. They performed Wright and Coombs tests by incubating patient serum at serial dilutions (>1:20) at 37°C for 48–72 hours; titers of >1:160 were considered diagnostically notable. Clinicians processed blood cultures by using BD BACTEC Plus Aerobic/F and Peds Plus/F bottles in the BD BACTEC 9120 automated system (BD). They confirmed positive cultures by using matrix-assisted laser desorption/ionization time-of-flight mass spectrometry with the MALDI Biotyper Microflex LT system (Bruker, https://www.bruker.com).

### Molecular Detection of *A. capra* DNA

We performed nested PCR targeting the *gltA* gene to detect *A. capra* DNA in EDTA-treated blood samples. We extracted genomic DNA by using the GeneAll Exgene Clinic SV kit (GeneAll, https://geneall.com), according to the manufacturer’s protocol. In the first round of PCR, we amplified 1031 bp of the *gltA* gene, and in the nested PCR step, we amplified 594 bp fragments of the *gltA* gene by using previously described primers (Table 1). We adapted PCR protocols from previously published methods (*20*,*24*). We included positive and negative controls in each PCR run to ensure accuracy and specificity.

**Table 1:** Primers used for amplification of the *gltA* and *groEL* genes of *Anaplasma capra* recovered from patients with anaplasmosis, Turkey

Target gene	Primer name	Primer sequence, 5′ → 3′	Amplified product size, bp	Reference
*gltA*	Outer-f	GCGATTTTAGAGTGYGGAGATTG	1,031	(*19*)
	Outer-r	TACAATACCGGAGTAAAAGTCAA		
	Inner-f	TCATCTCCTGTTGCACGGTGCCC	594	(*23*)
	Inner-r	CTCTGAATGAACATGCCCACCCT		
*groEL*	Outer-f	GCG AGG CGT TAG ACA AGT CCATT	1,129	(*19*)
	Outer-r	TCC AGA GAT GCA AGC GTG TATAG		
	Inner-f	TGA AGA GCA TCA AAC CCG AAG	874	(*23*)
	Inner-r	CTG CTC GTG ATG CTA TCG G		

### Genotyping of *A. capra* Strains

We obtained partial sequences of the *gltA* and *groEL* genes from all PCR-positive samples by using various primers (Table 1). We analyzed the nucleotide sequences by using BLAST (https://blast.ncbi.nlm.nih.gov) to determine their similarity to previously published *A. capra* sequences in GenBank. We submitted all the sequences generated to GenBank and obtained accession numbers for each sequence. We constructed phylogenetic trees for *A. capra gltA* and *groEL* sequences by using the maximum-likelihood method in MEGA 11 software (*25*). We determined the best-fit evolutionary model by using the Find Best-Fit Substitution Model tool in MEGA 11. We selected the Kimura 2-parameter model with gamma-distributed rates and invariant sites for the *gltA* gene (*26*), and we used the Tamura-Nei (TN93) model with gamma distribution among sites for the *groEL* gene (*27*). We performed bootstrap analysis with 1,000 replicates to assess tree robustness.

### Data Collection and Statistical Methods

We retrospectively extracted epidemiologic, clinical, laboratory, and outcome data from the electronic medical records of SCUH and entered the data into Microsoft Excel (Microsoft, https://www.microsoft.com). Collected data included patient demographics, symptoms, physical findings, laboratory values, and outcomes (fatal vs. nonfatal). Because this was a descriptive study, we did not perform inferential statistical analyses. We have presented our results as raw counts and percentages where applicable.

### Ethics Approval

The study was conducted in accordance with the Declaration of Helsinki and was approved by the research ethics committee of Sivas Cumhuriyet University (protocol no. 2022–12/16). Written informed consent was obtained from all participants.

## Results

### Diagnosis of CCHF, *A. capra* Infection, and Brucellosis

A total of 263 patients with suspected CCHF were enrolled in the study (100 female and 163 male). Among them, 206 (78.3%) patients were confirmed to have CCHF, diagnosed via CCHFV qRT-PCR (n = 197) or CCHFV IgM serology (n = 9). Nested PCR targeting a 594-bp fragment of the *A. capra gltA* gene identified 6 patients (2.28%) as positive for *A. capra.* All 6 patients were also positive for the *groEL* gene. Two patients were found to have co-infections with both *A. capra* and CCHFV. One patient had a dual infection with *A. capra* and *Brucella* sp., which was confirmed by positive blood culture. 

Among the *A. capra*-positive patients without co-infections, we performed an extended diagnostic workup for alternative infectious, autoimmune, and inflammatory etiologies, including viral hepatitis panels, HIV, COVID-19, Epstein-Barr virus, cytomegalovirus, *Rickettsia conorii*, Q fever, autoimmune antibodies (antinuclear, dsDNA, antineutrophil cytoplasmic, cyclic citrullinated peptide, rheumatoid factor, anticardiolipin), antistreptolysin O titer, and thyroid function tests. All test results were negative or unremarkable.

### Demographics and Characteristics of *A. capra* Infection

We summarized demographic, clinical, and laboratory characteristics of the 6 patients with anaplasmosis (Table 2). Patients were 30–83 years of age; 5 were female and 1 was male. Three (50%) patients reported a history of tick exposure. All 6 were residents in rural areas of Sivas (n = 5) and Tokat (n = 1) provinces (Figure 1). Common symptoms were fatigue (100%, n = 6), musculoskeletal pain (66.7%, n = 4), headache (66.7%, n = 4), and fever history (66.7%, n = 4). Physical examination revealed splenomegaly in 2 patients (33.3%) and current fever (38.3°C) in 1 patient (16.7%). No bleeding manifestations were observed in any of the cases. Leukopenia was observed in 3 patients (50%, n = 3), including 1 co-infected with *Brucella *sp. (patient 4) and another with CCHFV. Thrombocytopenia was observed in 4 patients (66.7%, n = 4), 2 of whom had co-infection with CCHFV and 1 with *Brucella *sp. (Table 3). One patient who was co-infected with CCHFV died (Table 2).

**Table 2:** Demographic characteristics, clinical symptoms, and physical findings of patients diagnosed with anaplasmosis caused by *Anaplasma capra*, Turkey*

Characteristic	Patient 1	Patient 2	Patient 3	Patient 4	Patient 5	Patient 6
Infectious agent	*A. capra* and CCHFV	*A. capra*	*A. capra*	*A. capra* and *Brucella* sp.	*A. capra* and CCHFV	*A. capra*
Age, y/sex	83/F	30/F	39/F	60/F	48/M	58/F
History of tick bite	No	Unknown	Yes	Unknown	Yes	Yes
Symptoms						
Weakness	Yes	Yes	Yes	Yes	Yes	Yes
Musculoskeletal pain	Yes	No	Yes	No	Yes	Yes
History of fever	Yes	No	No	Yes	Yes	Yes
Headache	No	Yes	No	Yes	Yes	Yes
Nausea and/or vomiting	Yes	No	No	No	No	No
Signs						
Splenomegalia	Yes	No	No	Yes	No	No
Fever, >38°C	Yes	No	No	No	No	No
Bleeding events†	No	No	No	No	No	No
Petechia	No	No	No	No	No	No
Hypotension, <90 mm Hg	Yes	No	No	No	No	No
Ecchymosis	Yes	No	No	No	No	No
Confusion	Yes	No	No	No	No	No
Co-infection	CCHFV	No	No	Brucellosis	CCHFV	No
Fatal outcome	Yes	No	No	No	No	No

*CCHFV, Crimean-Congo hemorrhagic fever virus. †At least 1 of epistaxis, bleeding gum, hemoptysis, hematuria, melena, and vaginal bleeding other than menstrual cycle.

**Figure 1:**
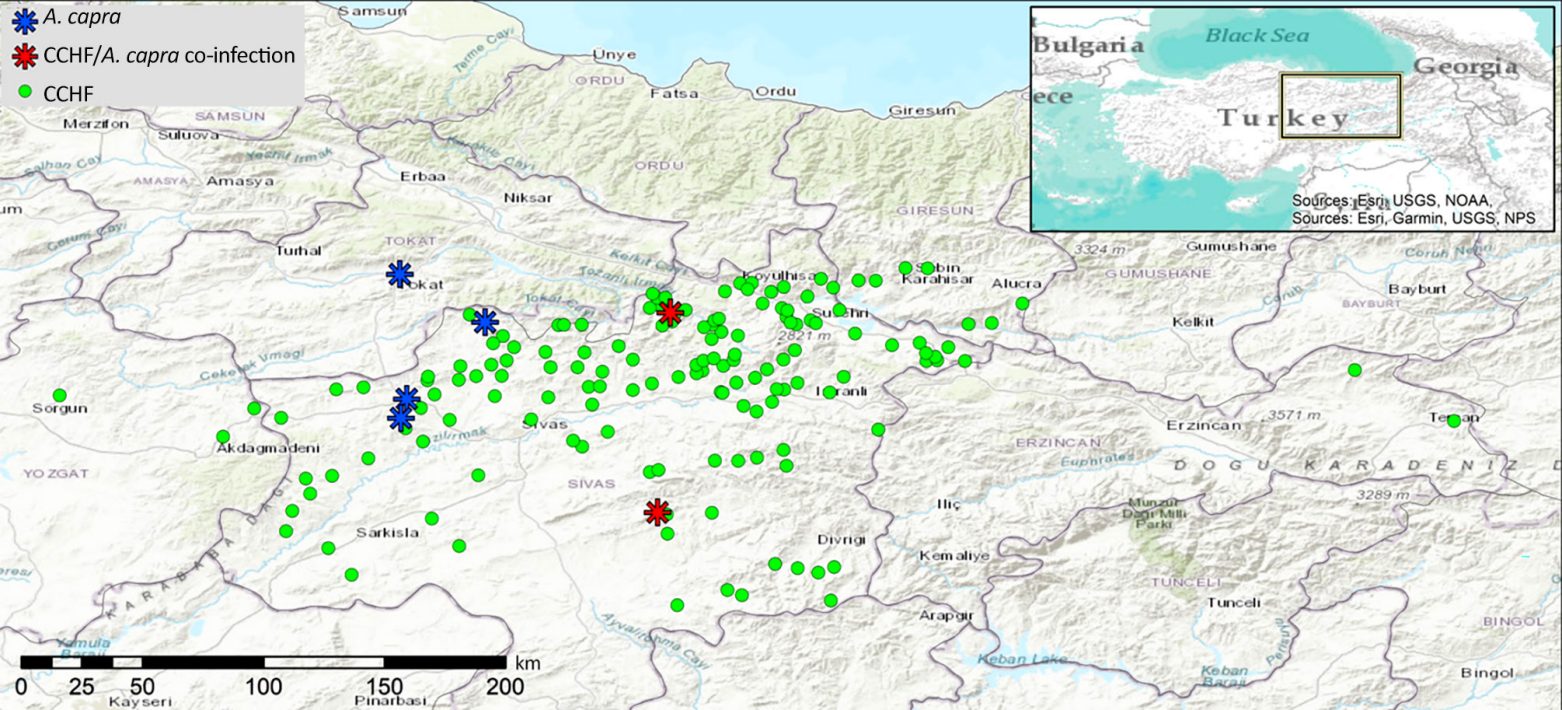
Geographic distribution of patients with suspected or confirmed CCHF in rural areas of Sivas, Tokat, Giresun, Yozgat, Erzincan, and Ordu Provinces, Turkey, 2022. Green dots represent confirmed CCHF patients (n = 206), blue asterisks represent *Anaplasma capra*–only cases, red asterisks represent *A. capra* and CCHF co-infections. Inset shows study area within Turkey. Map reproduced with permission from Zati Vatansever. CCHF, Crimean-Congo hemorrhagic fever.

**Table 3:** Laboratory testing results of patients diagnosed with anaplasmosis caused by *Anaplasma capra*, Turkey*

**Characteristic**	**Patient 1**	**Patient 2**	**Patient 3**	**Patient 4**	**Patient 5**	**Patient 6**
**Infectious agent**	***A. capra* and CCHFV**	***A. capra***	***A. capra***	***A. capra* and *Brucella* sp.**	***A. capra* and CCHFV**	***A. capra***
**Laboratory testing (reference range)**						
** Leukocytes, cells/mm^3^ (4,000–10,500)**	3,280	1,750	5,490	2,770	5,640	4,690
** Platelets, 10^3^/mm^3 ^(150–450)**	20	46	207	74	87	158
** Hemoglobin, g/dL (13.5–18.0 [M], 12.5–16.0 [F])**	11.1	10.1	15.6	9.7	14.3	12.4
** AST, IU/L (0–40)**	1,549	78	28	219	362	27
** ALT, IU/L (0–40)**	476	103	29	95	216	20
** CPK, µg/mm^3 ^(0–190)**	4,386	69	297	210	658	63
** LDH, IU/L (135–225)**	2,303	182	240	895	586	237
** PT, seconds (8.4–10.6)**	21.4	11.4	8.47	10.4	9.32	8.44
** aPTT, seconds (23.6–30.6)**	75.8	34.2	24.8	29.5	24.6	25

*ALT, alanine aminotransferase; aPTT, activated partial thromboplastin time; AST, aspartate aminotransferase; CCHFV, Crimean-Congo hemorrhagic fever virus; CPK, creatine phosphokinase; LDH, lactate dehydrogenase; PT, prothrombin time.

### Antimicrobial Drug Treatment for *A. capra*

Because of the retrospective nature of our study, patients did not receive targeted treatment for anaplasmosis during hospitalization. One surviving patient co-infected with *Brucella* sp. was successfully treated with a 42-day regimen of oral rifampin and doxycycline. Among the remaining patients, 1 (co-infected with CCHFV) died without receiving doxycycline therapy. The other 4 survivors were contacted and invited for postdischarge doxycycline treatment; however, they did not return for follow-up.

### *A. capra* *gltA* and *groEL* Sequencing and Phylogenetic Analyses

We submitted the *gltA* and *groEL* gene sequences obtained from all 6 *A. capra*–positive patients to GenBank (accession nos. OQ819441–6 [*gltA*] and OQ819447–52 [*groEL*]). Nucleotide similarity analysis of the *gltA* sequences revealed 84.16%–100% identity with previously published *A. capra* sequences in GenBank. The sequences exhibited high similarity (98.11%–100%) to strains from cattle, sheep, goats, and wild animals across various geographic regions. In addition, our human *A. capra* sequences revealed 100% identity with *A. capra* strains previously detected in water buffalo (GenBank accession nos. ON783817–9) in Turkey, as well as with sequences from sheep (accession no. MW930534), goats (accession no. MW930535), red deer (accession no. MH084720), and swamp deer (accession no. MH084719) in France and sheep in Kyrgyzstan (accession nos. OM100820–40).

Alignment of the *gltA* sequences revealed 0–8 nucleotide substitutions compared with 58 *A. capra* sequences from domestic and wild animals. In contrast, comparisons with 158 *A. capra* sequences from humans, animals, and ticks showed greater genetic diversity (84.16%–88.05% similarity), with 65–67 nucleotide differences observed (Appendix Figure 1). The phylogenetic tree created on the basis of *gltA* sequences indicated that the sequences clustered into 2 distinct genotypes (Figure 2).

**Figure 2:**
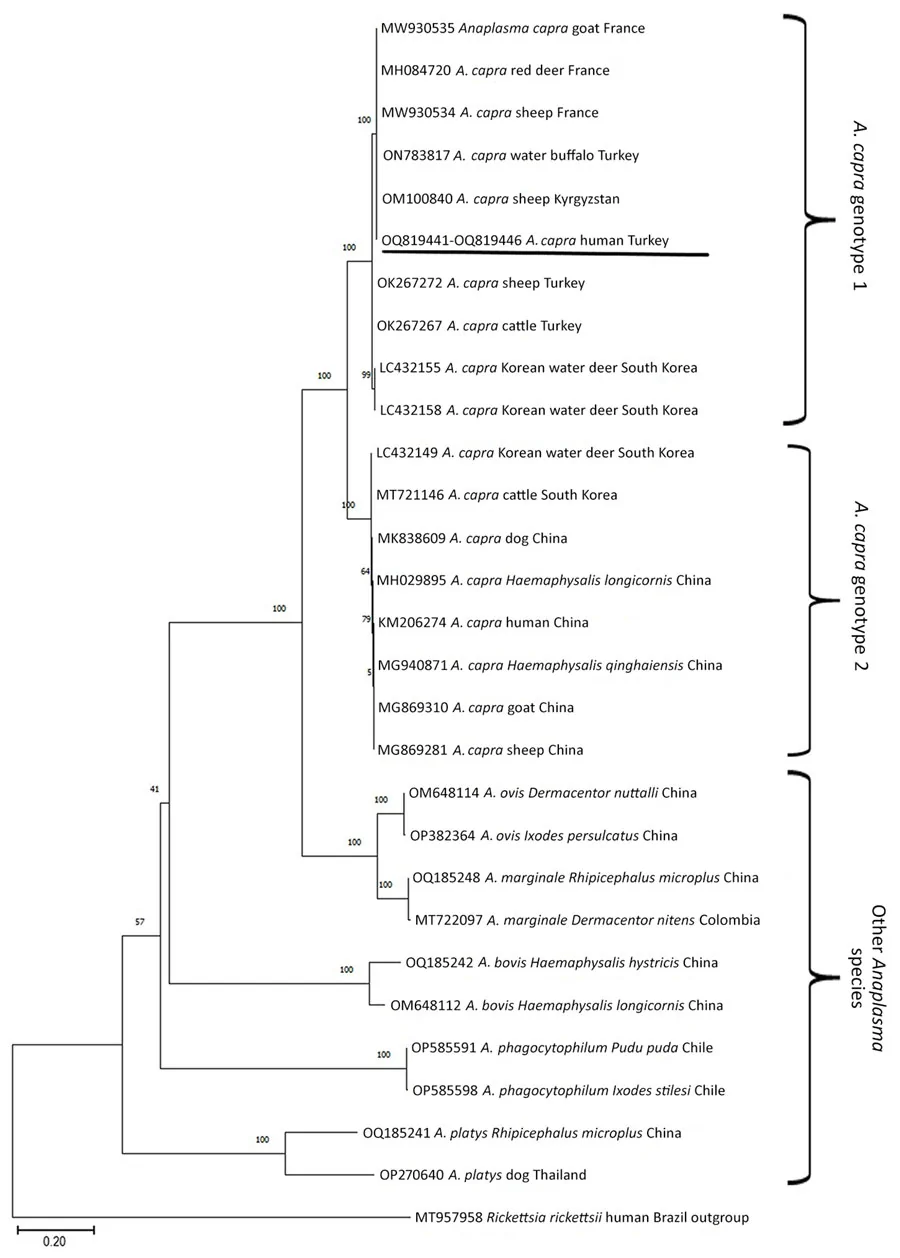
Phylogenetic tree of *gltA* gene sequences from *Anaplasma capra* genotype 1, genotype 2, and related species from patients with suspected or confirmed Crimean-Congo hemorrhagic fever, Turkey, 2022. We constructed the tree by using the maximum-likelihood method in MEGA 11 (*25*). *Rickettsia rickettsii* was used as the outgroup. Study sequences are underlined. GenBank accession numbers are provided. Scale bar represents nucleotide substitutions per site.

Similarly, the *groEL* gene sequences from the study samples showed 90.24%–100% identity with *A. capra* sequences in GenBank. We observed high nucleotide similarity (97.91%–100%) between our human *A. capra* sequences and those from water buffalo, sheep, goats, red deer, swamp deer, Korean water deer, roe deer, and ticks such as *Dermacentor everestianus* and *Haemaphysalis qinghaiensis.* Specifically, we noted 100% sequence identity with sequences from water buffalo in Turkey (GenBank accession nos. ON783820–2) and a sequence from a sheep in France (accession no. MW930531). Between our sequences and 26 closely related *A. capra* sequences, we found 0–17 nucleotide substitutions. However, we observed major differences (71–72 nucleotide substitutions) when sequences were compared with 138 *A. capra* sequences from domestic and wild animals, ticks, and humans (Appendix Figure 2). The phylogenetic tree we constructed from *groEL* sequences further supported the classification of sequences into 2 clades (Figure 3).

**Figure 3:**
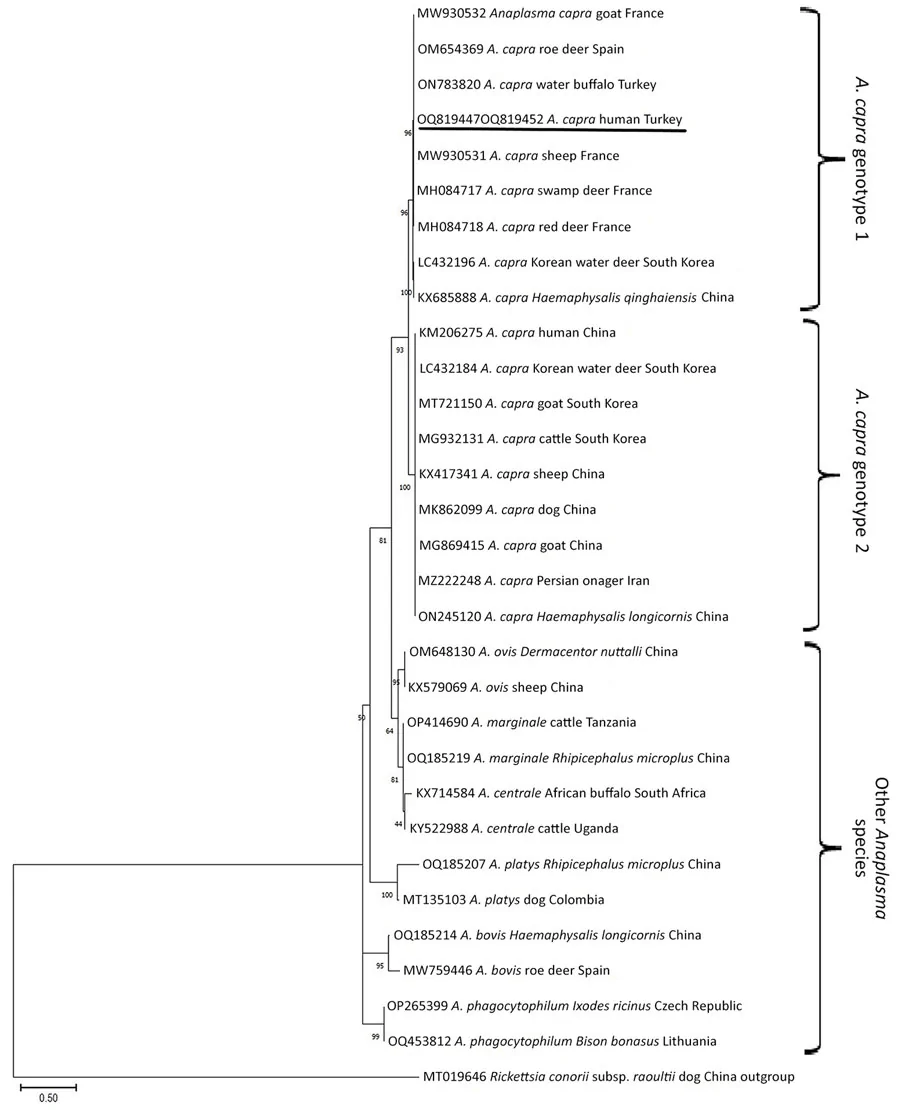
Phylogenetic tree of *groEL* gene sequences from *Anaplasma capra* genotype 1, genotype 2, and other *Anaplasma* species from patients with suspected or confirmed Crimean-Congo hemorrhagic fever, Turkey, 2022. We constructed the tree by using the maximum-likelihood method in MEGA 11 (*25*). *Rickettsia conorii* subspecies *raoultii* was used as the outgroup. Study sequences are underlined. GenBank accession numbers are provided. Scale bar represents nucleotide substitutions per site.

## Discussion

To date, multiple studies have demonstrated that *A. capra* can infect a wide range of hosts and might have a global distribution. The bacterium has been identified in various animal species, including goats, sheep (*28*), cattle (*29*), dogs (*30*), and yaks (*31*), and in multiple tick speciesm such as *Haemaphysalis qinghaiensis* (*31*), *H. longicornis* (*32*), *Rhipicephalus microplus* (*33*), *Dermacentor abaensis*, and *D. nuttalli* (*34*) in China. Beyond China, *A. capra* was first detected in cattle in Malaysia (*35*) and later in sheep in Sweden in 2018 (*36*), marking its identification in Europe. In 2021, it was also detected in cattle and ticks in Angola, establishing its presence in Africa (*37*). In Turkey, *A. capra* has been identified in cattle, sheep (*15*), and water buffalo (*18*). 

Most previous studies have focused on animal hosts and the molecular characterization of *A. capra.* However, we detected *A. capra* in 6 (2.28%) of 263 adult patients who were hospitalized for suspected CCHF. This study provides clinical evidence of *A. capra* infection in humans outside of China, confirming its zoonotic potential and geographic spread.

*Anaplasma* spp. bacteria are known to infect various blood cells, including monocytes, platelets, and granulocytes, where they replicate intracellularly (*14*). *A. capra* has been shown to infect erythrocytes under in vitro conditions, forming dark vacuolar inclusions or corpuscles in the cytoplasm (*38*). Clinically, CCHFV infection is known to range from mild illness to severe disease with multiorgan involvement and death. In contrast, human infections with *A. capra* have so far been associated with milder clinical manifestations, with no reported fatalities to date. Shared clinical and laboratory features include fever, malaise, headache, myalgia, and elevated hepatic enzyme levels, whereas leukopenia and thrombocytopenia have been reported only in a limited number of *A. capra* cases (*10*,*13*,*20*). In a previous study, 5 (17.9%) of 28 patients with *A. capra* infection were hospitalized for 11–21 days because of severe illness characterized by lymphadenopathy and elevated liver enzymes (*20*). Because of the shared transmission routes and overlapping clinical features, persons living in rural areas or involved in animal husbandry are at high risk for both CCHF and anaplasmosis. The CFR for anaplasmosis is typically low (<1%); doxycycline is the drug of choice for the treatment of anaplasmosis (*20*,*39*). One study reported that all 28 patients in their cohort were successfully treated with doxycycline with no fatalities (*20*). In our study, 1 of the 6 patients (16.7%) died; the patient was co-infected with CCHFV. 

Because CCHFV is well-established as causing death and ticks can harbor multiple pathogens (*6*), the specific contribution of *A. capra* infection to disease severity or mortality cannot be determined. However, we cannot determine whether *A. capra* and CCHFV and *A. capra* and *Brucella* sp. can be transmitted together by a single tick bite. This study and a previous one showed co-infections with CCHFV and other pathogens (*21*). Despite ticks having the potential capacity of *Brucella* spp. transmission, we found limited literature on tickborne brucellosis in humans (*40*). Because some patients are residents in rural areas and perform animal husbandry, they have risks for both CCHFV and *Brucella* spp. transmission. 

Our findings highlight the diagnostic challenges in patients with a history of tick exposure seeking care for nonspecific clinical manifestations. Therefore, diagnostic approaches should be guided by clinical evaluation and the limitations of available laboratory tests rather than focusing on a single etiologic agent. In such settings, empiric treatment with doxycycline might be considered when clinically indicated, particularly in endemic areas for tickborne diseases.

After the recognition of *A. capra*’s zoonotic potential, several molecular studies have investigated its genetic characteristics. Analyses of the 16S rRNA, *gltA*, and *groEL* genes have revealed substantial genetic variation across sequences (*15*–*18*,*20*). Research conducted in Turkey has identified 2 distinct genotypes of *A. capra*, genotype 1 and genotype 2. Although prior studies found no strong correlation between genotype and host species or geographic origin, all previously reported human cases in China were associated with genotype 2 (*15*,*17*,*18*). In our study, both *gltA* and *groEL* gene analyses confirmed that the human *A. capra* sequences clustered within genotype 1 (Appendix Figures 1,2). Although genotypes and 2 can be distinguished on the basis of gene sequencing, their roles in host specificity and disease manifestations remain unclear. Further studies are needed to better characterize those genotypes and clarify their clinical and epidemiologic significance.

The first limitation of this study is that serologic testing for *A. capra* was not performed because of the lack of validated and widely available assays, which limits our ability to support the molecular findings with serologic evidence. Second, the small number of cases restricts the generalizability of our findings. Finally, our study population might be subject to selection bias, because our inclusion criteria required the presence of leukopenia or thrombocytopenia (because of suspected CCHF).

In conclusion, the public health significance of tickborne pathogens continues to grow because of expanding interactions between humans and tick habitats. We found molecular evidence of *A. capra* infection in humans in Turkey. Our findings demonstrate that *A. capra* is circulating not only in domestic and wild animals but also in humans in the Middle Anatolia/Black Sea region, a known hyperendemic area for CCHF. Those results confirm the zoonotic potential of *A. capra* genotype 1 and highlight the need for further research to identify its biologic vectors and better understand its epidemiology in CCHF-endemic areas. Clinicians working in such regions should be aware of *A. capra* as a differential diagnosis in patients with a history of tick exposure and suspected CCHF, especially with negative CCHFV test results.

Appendix Additional information about detection of *Anaplasma capra* in patients with suspected Crimean-Congo hemorrhagic fever, Turkey, 2022.
