# Supplementary material for: Detection of Anaplasma capra in Patients with Suspected Crimean-Congo Hemorrhagic Fever, Turkey, 2022
Source: Emerg Infect Dis. 2026 Oct;32(10):1561–70. doi: 10.3201/eid3210.251049 (PMC13632829; doi:10.3201/eid3210.251049)
Supplement: Supplementary file 1 [file 25-1049-Techapp-s1.pdf]

Article DOI: https://doi.org/10.3201/eid3210.251049

EID cannot ensure accessibility for supplementary materials supplied by authors. Readers who have difficulty accessing supplementary content should contact the authors for assistance.

Detection of *Anaplasma capra* in Patients with Suspected Crimean-Congo Hemorrhagic Fever, Turkey, 2022

Appendix

Genbank Accession Numbers	Host	Country	Nucleotide Positions																																																																			
			174	182	184	186	187	188	198	219	225	231	237	247	255	258	267	270	273	282	287	291	297	303	311	312	318	333	334	339	348	351	360	366	369	379	384	394	404	411	423	430	475	478	481	492	516	537	549	552	558	561	567	572	573	579	582	585	588	591	601	606	619	636	642	651	654	665	666	669	671	690	696
OQ819441-OQ819446	Human	Türkiye	C	T	G	T	A	T	G	A	G	C	G	A	T	C	C	G	C	C	C	A	T	T	G	T	C	G	G	T	G	C	A	T	A	A	A	G	C	C	T	T	A	G	G	T	A	T	T	T	T	C	A	C	A	C	C	A	A	A	T	T	A	A	C	G	G	T	T	T	A	T	A
ON783817	Water buffalo	Türkiye	*	*	*	*	*	*	*	*	*	*	*	*	*	*	*	*	*	*	*	*	*	*	*	*	*	*	*	*	*	*	*	*	*	*	*	*	*	*	*	*	*	*	*	*	*	*	*	*	*	*	*	*	*	*	*	*	*	*	*	*	*	*	*	*	*	*	*	*	*	*	*
OM100840	Sheep	Kyrgyzstan	*	*	*	*	*	*	*	*	*	*	*	*	*	*	*	*	*	*	*	*	*	*	*	*	*	*	*	*	*	*	*	*	*	*	*	*	*	*	*	*	*	*	*	*	*	*	*	*	*	*	*	*	*	*	*	*	*	*	*	*	*	*	*	*	*	*	*	*	*	*	*
MW930534	Sheep	France	*	*	*	*	*	*	*	*	*	*	*	*	*	*	*	*	*	*	*	*	*	*	*	*	*	*	*	*	*	*	*	*	*	*	*	*	*	*	*	*	*	*	*	*	*	*	*	*	*	*	*	*	*	*	*	*	*	*	*	*	*	*	*	*	*	*	*	*	*	*	*
MW930535	Goat	France	*	*	*	*	*	*	*	*	*	*	*	*	*	*	*	*	*	*	*	*	*	*	*	*	*	*	*	*	*	*	*	*	*	*	*	*	*	*	*	*	*	*	*	*	*	*	*	*	*	*	*	*	*	*	*	*	*	*	*	*	*	*	*	*	*	*	*	*	*	*	*
MH084720	Red deer	France	*	*	*	*	*	*	*	*	*	*	*	*	*	*	*	*	*	*	*	*	*	*	*	*	*	*	*	*	*	*	*	*	*	*	*	*	*	*	*	*	*	*	*	*	*	*	*	*	*	*	*	*	*	*	*	*	*	*	*	*	*	*	*	*	*	*	*	*	*	*	*
OK267267	Cattle	Türkiye	–	–	–	–	–	–	*	*	*	*	*	*	*	*	*	*	*	*	*	*	*	C	*	C	*	*	*	*	*	*	*	*	*	*	*	*	*	*	*	*	*	*	*	*	*	*	*	*	*	*	*	*	*	*	*	*	*	*	*	*	*	*	*	*	*	*	*	*	*	*	*
OK267272	Sheep	Türkiye	–	–	*	*	G	*	*	*	*	*	*	*	*	*	*	*	*	*	*	*	*	C	*	C	*	*	*	*	*	*	*	*	*	*	*	*	*	*	*	*	*	*	*	*	*	*	*	*	*	*	*	*	*	*	*	*	*	*	*	*	*	*	*	*	*	*	*	*	*	*	*
LC432155	Water deer	South Korea	*	*	*	*	G	*	*	*	*	*	*	G	*	*	*	*	*	*	*	G	*	C	*	C	*	*	*	*	*	*	G	*	*	*	*	*	T	*	*	*	*	*	*	*	*	*	*	*	*	*	*	*	G	*	*	*	*	*	*	*	*	*	*	*	*	*	*	*	*	*	*
LC432158	Water deer	South Korea	*	*	*	*	G	*	*	*	*	*	*	G	*	*	*	*	*	*	*	G	*	C	*	C	*	*	*	*	*	*	G	*	*	*	*	*	T	*	*	*	*	*	*	*	*	*	*	*	*	*	*	*	G	*	*	*	*	*	*	*	*	*	*	*	*	*	*	*	*	*	*
MH029895	*H. longicornis*	China	T	C	T	C	G	C	T	G	A	G	A	*	G	T	G	A	T	T	T	*	C	C	C	C	T	A	A	C	A	T	*	C	G	G	C	A	T	T	C	C	G	A	A	C	G	C	C	C	C	T	G	T	*	T	T	T	G	G	G	C	G	G	T	A	T	C	C	C	G	C	C
KM206274	Human	China	T	C	T	C	G	C	T	G	A	G	A	*	G	T	G	A	T	T	T	*	C	C	C	C	T	A	A	C	A	T	*	C	G	G	C	A	T	T	C	C	G	A	A	C	G	C	C	C	C	T	G	T	*	T	T	T	G	G	G	C	G	G	T	A	T	C	C	C	G	C	C
MG940871	*H. qinghaiensis*	China	T	C	T	C	G	C	T	G	A	G	A	*	G	T	G	A	T	T	T	*	C	C	C	C	T	A	A	C	A	T	*	C	G	G	C	A	T	T	C	C	G	A	A	C	G	C	C	C	C	T	G	T	*	T	T	T	G	G	G	C	G	G	T	A	T	C	C	C	G	C	C
MG869310	Goat	China	T	C	T	C	G	C	T	G	A	G	A	*	G	T	G	A	T	T	T	*	C	C	C	C	T	A	A	C	A	T	*	C	G	G	C	A	T	T	C	C	G	A	A	C	G	C	C	C	C	T	G	T	*	T	T	T	G	G	G	C	G	G	T	A	T	C	C	C	G	C	C
MG869281	Sheep	China	T	C	T	C	G	C	T	G	A	G	A	*	G	T	G	A	T	T	T	*	C	C	C	C	T	A	A	C	A	T	*	C	G	G	C	A	T	T	C	C	G	A	A	C	G	C	C	C	C	T	G	T	*	T	T	T	G	G	G	C	G	G	T	A	T	C	C	C	G	C	C
MK838609	Dog	China	T	C	T	C	G	C	T	G	A	G	A	*	G	T	G	A	T	T	T	*	C	C	C	C	T	A	A	C	A	T	*	C	G	G	C	A	T	T	C	C	G	A	A	C	G	C	C	C	C	T	G	T	*	T	T	T	G	G	G	C	G	G	T	A	T	*	C	C	G	C	C
MT721146	Cattle	South Korea	T	C	T	C	G	C	T	G	A	G	A	*	G	T	G	A	T	T	T	*	C	C	C	C	T	A	A	C	A	T	*	C	G	G	C	A	T	T	C	C	G	A	A	C	G	C	C	C	C	T	G	T	*	T	T	T	*	G	G	C	G	G	T	A	T	*	C	C	G	C	C
LC432149	Water deer	South Korea	T	C	T	C	G	C	T	G	A	G	A	*	G	T	G	A	T	T	T	*	C	C	C	C	T	A	A	C	A	T	*	C	G	G	C	A	T	T	C	C	G	A	A	C	G	C	C	C	C	T	G	T	*	T	T	T	*	G	G	C	G	G	T	A	T	*	C	C	G	C	C

Abbreviations: Nucleotides C, Cytosine; T, Thymine; G, Guanine; A, Adenine. * Asterisks show the conserved nucleotide positions.

Appendix Figure 1. Nucleotide differences table with regard to partial nucleotide sequences of the *Anaplasma capra gltA* gene.

Nucleotide Positions

Genbank Accession Numbers	Host	Country	191	200	206	233	239	242	248	251	272	284	287	290	320	326	341	344	350	353	356	360	366	368
OQ819447-OQ819452	Human	Türkiye	T	C	T	T	T	T	A	C	C	G	T	A	T	G	G	C	G	A	G	G	C	A
ON783820	Water buffalo	Türkiye	*	*	*	*	*	*	*	*	*	*	*	*	*	*	*	*	*	*	*	*	*	*
MW930531	Sheep	France	*	*	*	*	*	*	*	*	*	*	*	*	*	*	*	*	*	*	*	*	*	*
MW930532	Goat	France	-	-	-	-	-	-	-	-	-	-	-	-	-	-	*	*	*	*	*	*	*	*
OM654369	Roe deer	Spain	*	*	*	*	*	*	*	*	*	*	*	*	*	*	*	*	*	*	*	*	*	*
MH084717	Swamp deer	France	*	*	*	*	*	*	*	*	*	*	*	*	*	*	*	*	*	*	*	*	*	*
MH084718	Red deer	France	*	*	*	*	*	*	*	*	*	*	*	*	*	*	*	*	*	*	*	*	*	*
LC432196	Water deer	South Korea	*	*	*	*	*	*	*	*	*	*	C	*	*	*	A	*	A	*	*	*	*	G
KX685888	*H. qinghaiensis*	China	*	*	C	*	*	*	*	*	*	*	*	*	*	*	*	*	A	*	*	T	*	G
KM206275	Human	China	C	T	*	C	C	C	G	T	T	T	*	G	C	A	*	T	A	C	A	*	T	G
MZ222248	Persian onager	Iran	C	T	*	C	C	C	G	T	T	T	*	G	C	A	*	T	A	C	A	*	T	G
MG869415	Goat	China	C	T	*	C	C	C	G	T	T	T	*	G	C	A	*	T	A	C	A	*	T	G
ON245120	*H. longicornis*	China	C	T	*	C	C	C	G	T	T	T	*	G	C	A	*	T	A	C	A	*	T	G
KX417341	Sheep	China	C	T	*	C	C	C	G	T	T	T	*	G	C	A	*	T	A	C	A	*	T	G
LC432184	Water deer	South Korea	C	T	*	C	C	C	G	T	T	T	*	G	C	A	*	T	A	C	A	*	T	G
MG932131	Cattle	South Korea	C	T	*	C	C	C	G	T	T	T	*	G	C	A	*	T	A	C	A	*	T	G
MK862099	Dog	China	C	T	*	C	C	C	G	T	T	T	*	G	C	A	*	T	A	C	A	*	T	G
MT721150	Goat	South Korea	C	T	*	C	C	C	G	T	T	T	*	G	C	A	*	T	A	C	A	*	T	G

Genbank Accession Numbers	Host	Country	383	391	392	410	422	431	437	440	443	445	473	476	482	485	491	497	503	512	533	536	563	584
OQ819447-OQ819452	Human	Türkiye	T	C	A	A	T	C	G	T	G	A	T	G	G	C	T	C	G	A	A	G	C	C
ON783820	Water buffalo	Türkiye	*	*	*	*	*	*	*	*	*	*	*	*	*	*	*	*	*	*	*	*	*	*
MW930531	Sheep	France	*	*	*	*	*	*	*	*	*	*	*	*	*	*	*	*	*	*	*	*	*	*
MW930532	Goat	France	*	*	*	*	*	*	*	*	*	*	*	*	*	*	*	*	*	*	*	*	*	*
OM654369	Roe deer	Spain	*	*	*	*	*	*	*	*	*	*	*	*	*	*	*	*	*	*	*	*	*	*
MH084717	Swamp deer	France	*	*	*	*	*	*	*	*	*	*	*	*	*	A	*	*	*	*	*	*	*	*
MH084718	Red deer	France	*	*	*	*	*	*	*	*	*	*	*	*	*	A	*	*	*	*	*	*	*	*
LC432196	Water deer	South Korea	*	*	*	*	*	*	A	C	*	*	*	*	*	A	*	*	A	G	*	*	*	*
KX685888	*H. qinghaiensis*	China	*	*	*	*	*	*	A	C	*	*	*	*	*	A	*	*	A	G	*	*	*	*
KM206275	Human	China	C	T	G	G	C	A	*	C	A	*	C	A	A	G	C	A	*	G	C	A	T	T
MZ222248	Persian onager	Iran	C	T	G	G	C	A	*	C	A	*	C	A	A	G	C	A	*	G	C	A	T	T
MG869415	Goat	China	C	T	G	G	C	A	*	C	A	*	C	A	A	G	C	A	*	G	C	A	T	T
ON245120	*H. longicornis*	China	C	T	G	G	C	A	*	C	A	*	C	A	A	G	C	A	*	G	C	A	T	T
KX417341	Sheep	China	C	T	G	G	C	A	*	C	A	*	C	A	A	G	C	A	*	G	C	A	T	T
LC432184	Water deer	South Korea	C	T	G	G	C	A	*	C	A	*	C	A	A	G	C	A	*	G	C	A	T	T
MG932131	Cattle	South Korea	C	T	G	G	C	A	*	C	A	*	C	A	A	G	C	A	*	G	C	A	T	T
MK862099	Dog	China	C	T	G	G	C	A	*	C	A	G	C	A	A	G	C	A	*	G	C	A	T	T
MT721150	Goat	South Korea	C	T	G	G	C	A	*	C	A	*	C	A	A	G	C	A	*	G	C	A	T	T

Genbank Accession Numbers	Host	Country	587	599	602	617	635	656	662	665	673	687	692	710	720	728	740	743	749	764	770	791	794	800
OQ819447-OQ819452	Human	Türkiye	C	C	T	A	T	A	C	G	A	C	A	C	C	C	G	G	A	C	C	T	A	A
ON783820	Water buffalo	Türkiye	*	*	*	*	*	*	*	*	*	*	*	*	*	*	*	*	*	*	*	*	*	*
MW930531	Sheep	France	*	*	*	*	*	*	*	*	*	*	*	*	*	*	*	*	*	*	*	*	*	*
MW930532	Goat	France	*	*	*	*	*	*	*	*	*	*	*	*	*	*	*	*	*	*	*	*	*	*
OM654369	Roe deer	Spain	*	*	*	*	*	*	*	*	*	*	*	*	*	*	*	*	*	*	*	*	*	*
MH084717	Swamp deer	France	*	*	*	*	*	*	*	*	*	*	*	*	*	*	*	*	*	*	*	*	*	*
MH084718	Red deer	France	*	*	*	*	*	*	*	*	*	*	*	*	*	*	*	*	*	*	*	*	*	*
LC432196	Water deer	South Korea	*	*	*	*	*	*	*	*	*	*	*	*	*	*	*	*	*	*	*	*	*	*
KX685888	*H. qinghaiensis*	China	*	*	*	G	*	*	*	*	*	T	*	*	*	*	*	*	*	*	*	*	*	G
KM206275	Human	China	T	T	C	*	C	G	T	T	G	*	G	T	T	T	A	A	G	T	G	C	G	*
MZ222248	Persian onager	Iran	T	T	C	*	C	G	T	T	G	*	G	T	T	T	A	A	G	T	G	C	G	*
MG869415	Goat	China	T	T	C	*	C	G	T	T	G	*	G	T	T	T	A	A	G	T	G	C	G	*
ON245120	*H. longicornis*	China	T	T	C	*	C	G	T	T	G	*	G	T	T	T	A	A	G	T	G	C	G	*
KX417341	Sheep	China	T	T	C	*	C	G	T	T	G	*	G	T	T	T	A	A	G	T	G	C	G	*
LC432184	Water deer	South Korea	T	T	C	*	C	G	T	T	G	*	G	T	T	T	A	A	G	T	A	C	G	*
MG932131	Cattle	South Korea	T	T	C	*	C	G	T	T	G	*	G	T	T	T	A	A	G	T	A	C	G	*
MK862099	Dog	China	T	T	C	*	C	G	T	T	G	*	G	T	T	T	A	A	G	T	G	C	G	*
MT721150	Goat	South Korea	T	T	C	*	C	G	T	T	G	*	G	T	T	T	A	A	G	T	A	C	G	*

Genbank Accession Numbers	Host	Country	821	830	845	848	857	860	872	874	878	881	887	899	923	929	944	953	959	962	974	989
OQ819447-OQ819452	Human	Türkiye	C	G	T	C	C	G	T	G	C	A	T	A	G	C	G	C	A	A	G	A
ON783820	Water buffalo	Türkiye	*	*	*	*	*	*	*	*	*	*	*	*	*	*	*	*	*	*	*	*
MW930531	Sheep	France	*	*	*	*	*	*	*	*	*	*	*	*	*	*	*	*	*	*	*	*
MW930532	Goat	France	*	*	*	*	*	*	*	*	*	*	*	*	*	*	*	*	*	*	*	*
OM654369	Roe deer	Spain	-	-	-	-	-	-	-	-	-	-	-	-	-	-	-	-	-	-	-	-
MH084717	Swamp deer	France	*	*	*	*	*	*	*	*	*	*	*	*	*	*	*	*	*	*	*	*
MH084718	Red deer	France	*	*	*	*	*	*	*	*	*	*	*	*	*	*	*	*	*	*	*	*
LC432196	Water deer	South Korea	*	*	*	T	*	*	*	*	T	*	*	G	*	*	*	T	G	*	A	*
KX685888	*H. qinghaiensis*	China	*	*	*	T	*	*	*	*	T	*	*	G	*	*	*	T	*	*	A	*
KM206275	Human	China	T	A	G	T	T	A	C	A	*	G	C	*	A	T	A	*	*	G	A	G
MZ222248	Persian onager	Iran	T	A	G	T	T	A	C	A	*	G	C	*	A	T	A	*	*	G	A	G
MG869415	Goat	China	T	A	G	T	T	A	C	A	*	G	C	*	A	T	A	*	*	G	A	G
ON245120	*H. longicornis*	China	T	A	G	T	T	A	C	A	*	G	C	*	A	T	A	*	*	G	A	G
KX417341	Sheep	China	T	A	G	T	T	A	C	A	*	G	C	*	A	T	A	*	*	G	A	G
LC432184	Water deer	South Korea	T	A	G	T	T	A	C	A	*	G	C	*	A	T	A	*	*	G	A	G
MG932131	Cattle	South Korea	T	A	G	T	T	A	C	A	*	G	C	*	A	T	A	*	*	G	A	G
MK862099	Dog	China	T	A	G	T	T	A	C	A	*	G	C	*	A	T	A	*	*	G	A	G
MT721150	Goat	South Korea	T	A	G	T	T	A	C	A	*	G	C	*	A	T	A	*	*	G	A	G

Abbreviations: Nucleotides C, Cytosine; T, Thymine; G, Guanine; A, Adenine. * Asterisks show the conserved nucleotide positions.

Appendix Figure 2. Nucleotide differences table according to partial nucleotide sequences of the *Anaplasma capra groEL* gene.
